# P-1311. Community-associated carbapenem-resistant gram-negative bacilli infection in Japan

**DOI:** 10.1093/ofid/ofaf695.1499

**Published:** 2026-01-11

**Authors:** Yuya Kawamoto, Kohei Uemura, Yasufumi Matsumara, Ryota Hase, Hideaki Kato, Takashi Matono, Naoya Itoh, Takehiro Hashimoto, Go Yamamoto, Momoko Mawatari, Takeya Tsutsumi, Tetsuya Suzuki, Koji Ohyama, Masahiro Suzuki, Aki Sakurai, Kayoko Hayakawa, David van Duin, Norio Ohmagari, Yohei Doi, Sho Saito

**Affiliations:** Fujita Health University School of Medicine, Toyoake, Aichi, Japan; The University of Tokyo, Bunkyo-ku, Tokyo, Japan; Kyoto University Graduate School of Medicine, Kyoto, Kyoto, Japan; Japanese Red Cross Narita Hospital, Narita-shi, Chiba, Japan; Yokohama City University Hospital, yokohama-shi, Kanagawa, Japan; Saga University Hospital, Saga, Saga, Japan; Nagoya City University, Nagoya, Aichi, Japan; Oita University Hospital, Yufu-shi, Oita, Japan; Osaka University Graduate School of Medicine, Suita, Osaka, Japan; Department of Infectious Diseases, Japanese Red Cross Medical Center, Tokyo, Japan, Shibuya, Tokyo, Japan; The University of Tokyo Hospital, Bunkyo-ku, Tokyo, Japan; University of Yamanashi Hospital, Chuo, Yamanashi, Japan; Department of Microbiology, Fujita Health University School of Medicine, Toyoake, Aichi, Japan; Fujita Health University School of Medicine, Toyoake, Aichi, Japan; University of Texas Health Science Center, McGovern Medical School, Houston, TX; Japan Institute for Health Security, National Center for Global Health and Medicine, Tokyo, Tokyo, Japan; University of North Carolina at Chapel Hill, Chapel Hill, NC; Japan Institute for Health Security, Shinjuku-ku, Tokyo, Japan; Fujita Health University, Aichi, Aichi, Japan; Japan Institute for Health Security, Shinjuku-ku, Tokyo, Japan

## Abstract

**Background:**

Carbapenem-resistant Gram-negative bacilli (CR-GNB) infections are associated with high mortality and increased healthcare costs. While certain resistant pathogens, such as extended-spectrum β-lactamase-producing organisms, are increasingly identified in the community, reports of community-associated CR-GNB infections remain limited, particularly in high-income countries like Japan.

**Table 1.**
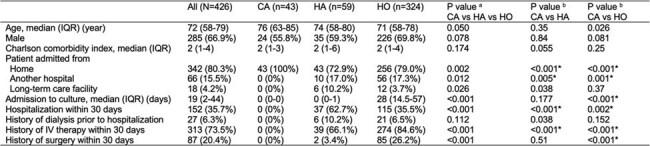
Demographic characteristics of patients with community-associated, healthcare-associated, and hospital-onset CR-GNB infections. CR-GNB, carbapenem-resistant Gram-negative bacilli; CA, community-associated; HA, healthcare-associated; HO, hospital-onset; IQR, interquartile range; IV, intravenous. a P values for comparisons among the three groups (CA, HA, HO) were calculated using the Kruskal–Wallis test for continuous variables and the chi-squared test for categorical variables. b Pairwise comparisons were performed using the Mann–Whitney U test for continuous variables and Fisher’s exact test for categorical variables. For multiple comparisons, a Bonferroni-corrected threshold of P <0.025 was considered statistically significant. Asterisks indicate statistical significance at the corrected threshold.

**Table 2.**
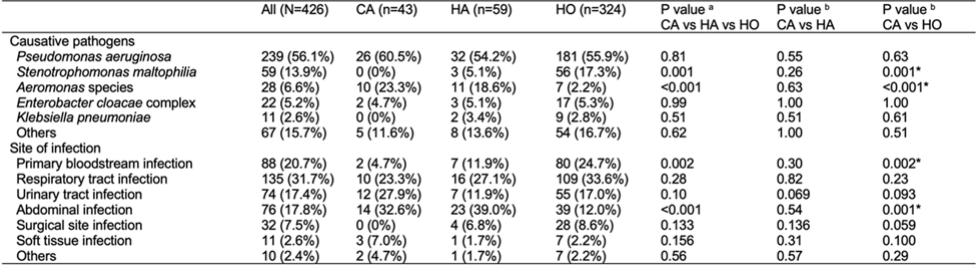
Causative pathogens and sites of infection among patients with community-associated, healthcare-associated, and hospital-onset CR-GNB infections. CR-GNB, carbapenem-resistant Gram-negative bacilli; CA, community-associated; HA, healthcare-associated; HO, hospital-onset. a P values for comparisons among the three groups (CA, HA, HO) were calculated using the Kruskal–Wallis test for continuous variables and the chi-squared test for categorical variables. b Pairwise comparisons were performed using the Mann–Whitney U test for continuous variables and Fisher’s exact test for categorical variables. For multiple comparisons, a Bonferroni-corrected threshold of P <0.025 was considered statistically significant. Asterisks indicate statistical significance at the corrected threshold.

**Methods:**

From April 2019 to March 2022, we prospectively enrolled patients with CR-GNB infections through the Multidrug-Resistant Organisms Clinical Research Network (MDR-net), comprising 13 tertiary care centers in Japan. We compared patient demographics, clinical characteristics, and outcomes across community-associated (CA), healthcare-associated (HA), and hospital-onset (HO) infections.

**Table 3.**
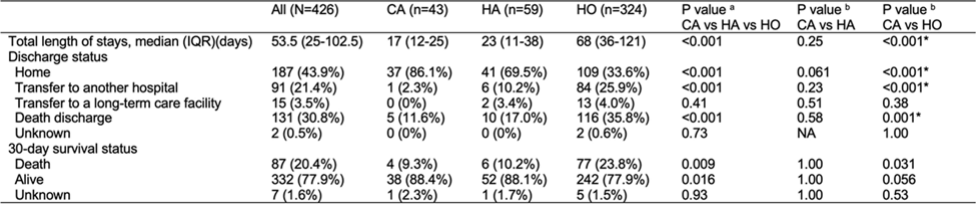
Clinical outcome of patients with community-associated, healthcare-associated, and hospital-onset CR-GNB infections. CR-GNB, carbapenem-resistant Gram-negative bacilli; CA, community-associated; HA, healthcare-associated; HO, hospital-onset. a P values for comparisons among the three groups (CA, HA, HO) were calculated using the Kruskal–Wallis test for continuous variables and the chi-squared test for categorical variables. b Pairwise comparisons were performed using the Mann–Whitney U test for continuous variables and Fisher’s exact test for categorical variables. For multiple comparisons, a Bonferroni-corrected threshold of P <0.025 was considered statistically significant. Asterisks indicate statistical significance at the corrected threshold. 95% confidence intervals for death-related outcomes by onset: Death discharge – CA: 11.6% (95% CI: 1.9–21.4), HA: 17.0% (7.3–26.6), HO: 35.8% (30.6–41.0); 30-day mortality – CA: 9.3% (0.5–18.1), HA: 10.2% (2.4–17.9), HO: 23.8% (19.1–28.4).

**Figure 1.**
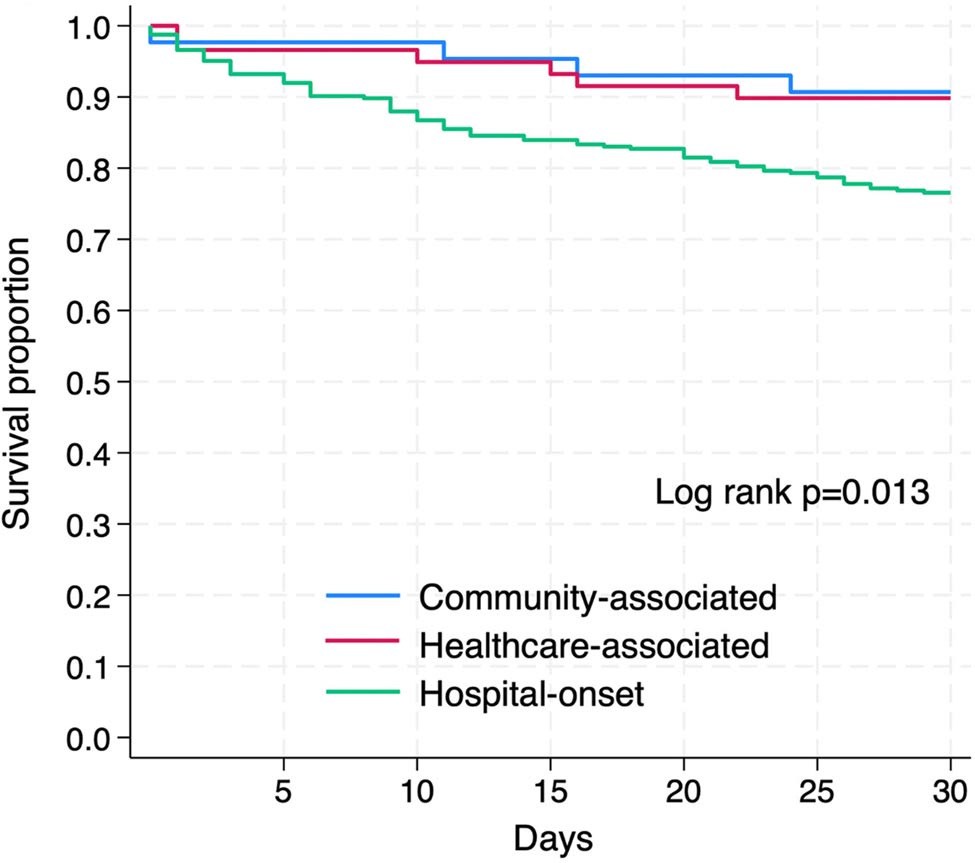
Kaplan–Meier survival curves showing patient days from the first positive culture to death for patients with community-associated, healthcare-associated, and hospital-onset CR-GNB infections. CR-GNB, carbapenem-resistant Gram-negative bacilli

**Results:**

Among 426 patients with CR-GNB infections (CA, n=43; HA, n=59; HO, n=324), most were elderly, with comparable Charlson Comorbidity Index scores across groups (median [IQR], 2 [1–4]; P = 0.17). *Pseudomonas aeruginosa* was the most frequently isolated pathogen in all groups. *Aeromonas* species were significantly more prevalent in CA and HA compared to HO (CA: 23.3%, HA: 18.6%, HO: 2.2%; P < 0.001 overall), whereas *Stenotrophomonas maltophilia* was predominantly isolated in HO (HO: 17.3%, HA: 5.1%, CA: 0%; P = 0.001 overall). Clinical outcomes differed significantly by infection type. Compared to CA, HO infections were associated with longer hospital stay (68 vs. 17 days, P < 0.001), lower discharge to home (33.6% vs. 86.1%, P < 0.001), and higher in-hospital mortality (35.8% vs. 11.6%, P = 0.001). Thirty-day mortality also differed, with Kaplan–Meier analysis showing the lowest survival in HO (log-rank P = 0.013). In contrast, HA outcomes were comparable to CA, with no significant differences in length of stay (23 vs. 17 days, P = 0.25), discharge to home (69.5% vs. 86.1%, P = 0.061), or in-hospital mortality (17.0% vs. 11.6%, P = 0.58). These findings suggest that poor outcomes in CR-GNB infections were mainly driven by HO cases.

**Conclusion:**

The clinical features and outcomes of CR-GNB infections differ markedly by onset, with HO infections associated with poorer outcomes compared to CA and HA infections.

**Disclosures:**

Yasufumi Matsumara, MD, PhD, Beckman Coulter: Research support for a collaborative project|Precision System Science: Research support for a collaborative project Takashi Matono, MD, PhD, FACP, Gilead Sciences: Honoraria|GSK: Honoraria|Meiji Seika Pharma: Honoraria|MSD: Honoraria|Pfizer: Honoraria Naoya Itoh, MD, DTM&H, PhD, Asahi Kasei Pharma Corporation: Honoraria|AstraZeneca K.K: Honoraria|BD Co, Ltd: Honoraria|bioMérieux Japan Ltd: Honoraria|Gilead Sciences Inc: Honoraria|GlaxoSmithKline: Honoraria|Meiji Seika Pharma Co, Ltd: Honoraria|MSD K.K: Honoraria|Pfizer;: Honoraria|shimadzu co ltd: Grant/Research Support|Shionogi Co, Ltd: Honoraria Tetsuya Suzuki, M.D., Ph.D., Meiji Seika Pharma: Honoraria David van Duin, MD, PhD, British Society for Antimicrobial Chemotherapy: Editor stipend|Merck: Advisor/Consultant|Merck: Grant/Research Support|Pfizer: Advisor/Consultant|Roche: Advisor/Consultant|Shionogi: Advisor/Consultant Yohei Doi, MD, PhD, GSK: Advisor/Consultant|Meiji Seika Pharma: Advisor/Consultant|Shionogi: Advisor/Consultant|Shionogi: Honoraria Sho Saito, Dr, Shionogi & Co., Ltd.: Grant/Research Support|SUNSTAR: Grant/Research Support

